# Pain management for medical and surgical termination of pregnancy between 13 and 24 weeks of gestation: a systematic review

**DOI:** 10.1111/1471-0528.16212

**Published:** 2020-04-03

**Authors:** E Jackson, N Kapp

**Affiliations:** ^1^ Ipas Chapel Hill NC USA

**Keywords:** Dilatation and evacuation, induced termination of pregnancy, medical termination of pregnancy, pain management, second trimester

## Abstract

**Background:**

High‐quality care for termination of pregnancy (TOP) requires pain to be effectively managed; however, practices differ, and the available guidelines do not specify optimal strategies.

**Objective:**

To guide providers in effective pain management for second‐trimester medical and surgical TOP.

**Search strategy:**

We searched PubMed, Cochrane and Embase databases, and the US National Library of Medicine clinical trials registry, from inception to the end of June 2019, and hand‐searched reference lists.

**Selection criteria:**

Trials comparing pain management strategies with no treatment, placebo or active interventions during induced medical or surgical TOP, occurring between 13 and 24 weeks of gestation, and reporting direct or indirect measures of pain.

**Data collection and analysis:**

Both authors summarised and systematically assessed the evidence and risk of bias using standard tools.

**Main results:**

We included seven medical and four surgical TOP studies, with 453 and 349 participants, respectively. The heterogeneity of interventions and outcomes prevented pooled analyses. Medical TOP: women receiving routine or continuous epidural analgesia experienced mild pain. The prophylactic use of nonsteroidal anti‐inflammatory drugs (NSAIDs) decreased pain (mean difference −0.5, *P* < 0.001) and additional opioid requirements (3.5 versus 7 mg, *P* = 0.04) compared with placebo/other treatment. Paracervical block was ineffective. No studies assessed intramuscular (IM)/intravenous (IV) opioid or nonpharmacological treatment. Surgical TOP: general anaesthesia/deep IV sedation alleviated pain. Nitrous oxide was ineffective. No studies assessed moderate IV sedation, IV/IM opioid, paracervical block without sedation, NSAID or nonpharmacological treatment.

**Conclusion:**

Based on limited data, regional analgesia and NSAIDs mitigated second‐trimester medical TOP pain; general anaesthesia/deep IV sedation alleviated surgical TOP pain.

**Tweetable abstract:**

Although women experience intense pain during second‐trimester termination of pregnancy, few data are available to inform their treatment.

## Tweetable abstract

Although women experience intense pain during second‐trimester termination of pregnancy, few data are available to inform their treatment.

## Introduction

Pain is a predictable feature of the process of uterine evacuation, whether the termination of pregnancy (TOP) occurs by the administration of medicines or by a transcervical procedure.^1^, ^2^, ^3^, ^4^, ^5^ Although most women who undergo induced TOP do so early in pregnancy, around 10% of women who need TOP globally have a gestation of >13 weeks.^6^, ^7^ TOP at later gestational ages increases the intensity and length of time that women experience pain, necessitating greater or different pain management strategies than are required in early pregnancy.^2^, ^8^

The purpose of pain management is to decrease discomfort, pain and possibly anxiety, with the lowest risk to a woman's health; it is an important component of the quality of TOP care services.^9^, ^10^ Despite this, clinical studies have not established the optimal regimen for pain management during induced TOPs occurring after 13 weeks of gestation. Pain is difficult to research. Most measures of pain are subjective and are highly variable between individuals, facilities often dictate the availability of analgesics or anaesthetics and practices vary between TOP providers, who commonly also provide obstetrical, surgical or general medicine services. Currently, international guidelines such as those issued by the World Health Organization or the Royal College of Obstetricians and Gynaecologists recommend that women be offered pain management strategies, without stipulating one or many regimens from which to choose.^11^, ^12^

To guide TOP programmes and providers in how best to provide pain management to their clients, we conducted a systematic review of pain management strategies for women undergoing induced TOP in the second trimester of pregnancy.

## Methods

We searched PubMed, Cochrane and Embase databases for all articles, unrestricted by language, published in peer‐reviewed journals, from database inception through to the end of June 2019, reporting studies of pain management strategies for medical and surgical TOP performed between 13 and 24 weeks of gestation. Search strategies were developed in consultation with a librarian skilled in systematic searching. Our PubMed search strategy (Appendix S1) used a combination of Medical Subject Headings (MeSH) terms and keywords; this search was adapted for the Cochrane and Embase databases. Additionally, we searched the US National Library of Medicine clinical trials database (ClinicalTrials.gov) using a keyword search (‘abortion’, ‘second trimester’ and ‘pain’) to identify completed studies that had not yet been published and reviewed reference lists from retrieved articles to identify additional reports. We did not search abstracts from scientific meetings.

One author (EJ) initially screened article titles and abstracts, removing those that clearly did not meet the review criteria; both authors (EJ and NK) independently reviewed the remaining abstracts and full texts for possible inclusion in the review. We anticipated finding few randomised controlled trials (RCTs) related to this topic. As such, in addition to RCTs, we included non‐randomised comparative trials and prospective and retrospective comparative cohort studies. We included studies assessing any pain management strategy, including non‐pharmacological pain management, for women undergoing either medical or surgical TOP for gestations of between 13 and 24 weeks. For medical TOP studies, we included studies reporting the use of a combined mifepristone and misoprostol regimen, misoprostol‐only regimen or regimens using gemeprost to induce TOP; we excluded studies using obsolete medical TOP regimens, such as ethacridine lactate, prostaglandin F2α, hypertonic saline or urea.

We excluded studies if we could not determine the gestational age range to be between 13 and 24 weeks. Where reports were not disaggregated by gestational age, we contacted the authors to request these data;^13^ if disaggregated data were not provided, studies were included if the average gestational age was within the specified range. We also contacted the authors of relevant studies listed in the ClinicalTrials.gov database that were reported to have completed recruitment, but that had not yet been published, and invited authors to contribute their data to the review. We excluded studies only recruiting women with pregnancies after 24 weeks of gestation, or studies treating only incomplete TOP, missed TOP or intrauterine fetal demise (i.e. postabortion care). We included studies with a mix of induced TOP and postabortion care patients if data related to induced TOP were disaggregated or if we were able to acquire disaggregated data from the study authors.

Our primary outcomes were measures assessing the effectiveness of pain management strategies. We included direct measures of pain, such as patient‐reported effectiveness, during or after medical or surgical TOP, and patient acceptability of and satisfaction with pain management, as well as indirect measures, such as the need for additional analgesia. We excluded studies that did not report a direct measure of pain. To assess the safety of pain management strategies, we examined TOP‐related and pain management‐related complications, adverse events and side effects, and in studies that used sedation or general anaesthesia, we assessed anaesthesia‐related complications, adverse events and side effects. For medical TOP studies, the time between induction to TOP and TOP success rates were also assessed.

One author (EJ) conducted an initial data extraction and risk‐of‐bias assessment using standard forms and the Cochrane Collaborative's Tool.^14^ All data assessment was reviewed and independently confirmed by the second author (NK); disagreements were resolved by consensus. We planned pooled analyses for each comparison where more than one study reported comparable pain management interventions and similar outcome measures. Where these conditions were not met, we planned a narrative synthesis of the results.

Patients were not involved in the planning or conduct of this systematic review. A core outcome set for TOP care is currently in development,^15^ but is not yet available at the time of this review. This work was paid for internally by Ipas, as part of a continuing effort to provide up‐to‐date, evidence‐based clinical guidance for TOP providers and programmes globally.^16^

## Results

**Figure 1:**
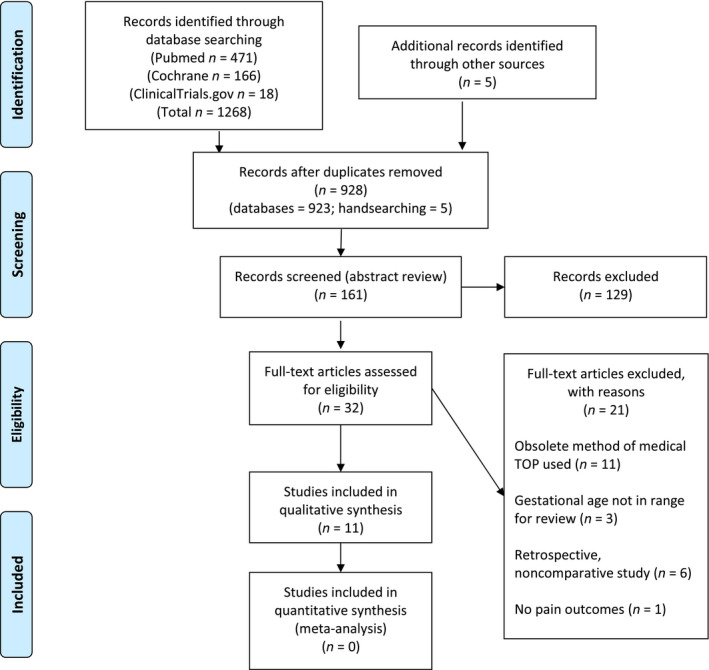
Preferred Reporting Items for Systematic Reviews and Meta‐Analysis (PRISMA) 2009 flow diagram.

Our search strategy yielded a total of 928 citations after removing duplicates. Eleven studies met the inclusion criteria: ten RCTs and one noncomparative trial.^17^ For the Preferred Reporting Items for Systematic Reviews and Meta‐Analysis (PRISMA) flow diagram, see Figure 1.^18^ All included studies exclusively reported findings from induced TOP. As a result of the heterogeneity of pain management strategies and reported outcomes, pooled analyses were not possible.

**Figure 2:**
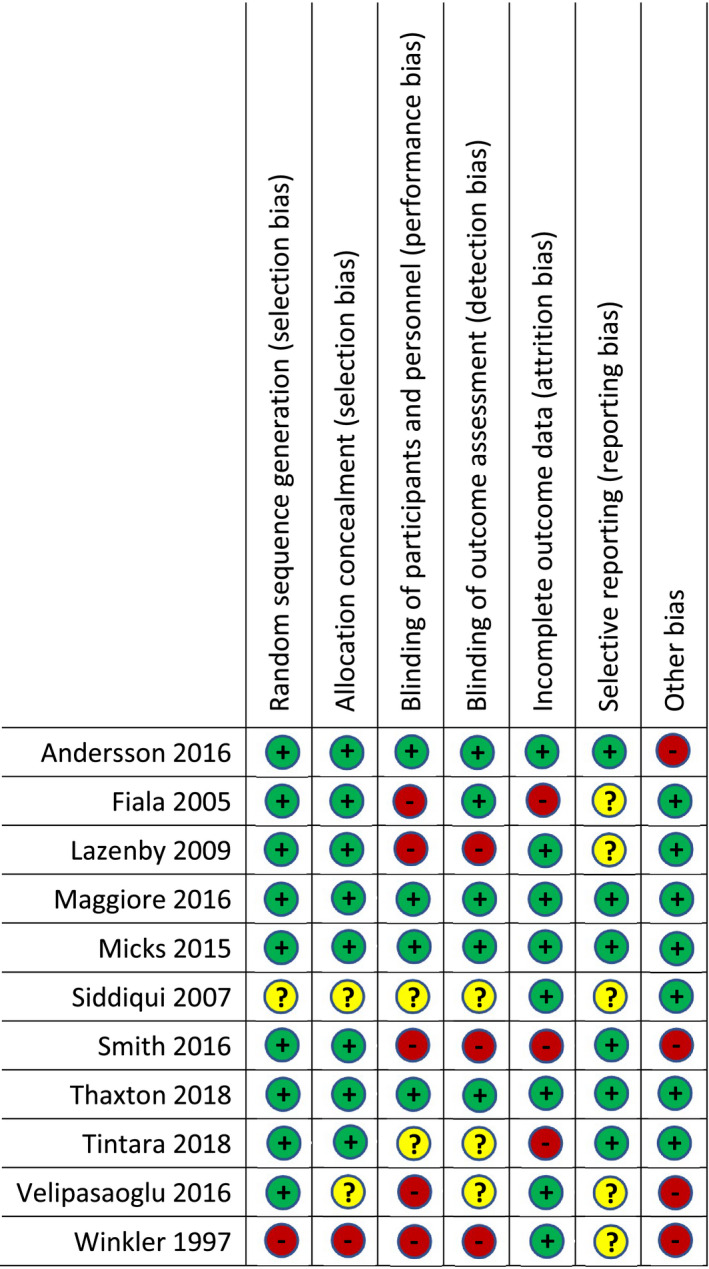
Summary risk of bias for all included studies in the review.

Seven studies reported results from medical TOP (Figure 2; see Tables 1 and Table S1) among 453 participants hailing from Europe (five studies), Canada (one study) and Thailand (one study).^17^, ^19^, ^20^, ^21^, ^22^ The mean gestational age ranged from 16 to 22 weeks. Indications for TOP were described in six studies: in one, all TOPs were performed for fetal indications;^17^ in another study, 80% were performed for fetal indications;^22^ in three studies, fewer than half of the TOPs were performed for fetal indications;^19^, ^21^, ^24^ and one study reported that all TOPs were ‘indicated,’ but did not specify those indications.^20^ TOP was induced with mifepristone plus misoprostol in two studies,^19^, ^21^ misoprostol alone was used in three studies,^20^, ^23^, ^24^ and gemeprost was used in two studies.^17^, ^22^ To report women's pain, six studies used an 11‐point visual analogue scale (VAS),^17^, ^19^, ^20^, ^21^, ^22^, ^24^ and one used an 11‐point verbal scale;^23^ two studies reported participant satisfaction with pain management using a visual or verbal scale.^22^, ^23^ Five studies reported the need for additional opioid pain medications for the participants beyond those of the study interventions;^17^, ^19^, ^20^, ^21^, ^24^ in contrast, studies using regional analgesia reported the overall consumption of pain medications or the need for additional anxiolytic medication.^22^, ^23^

**Table 1:** Included studies of pain management for medical termination of pregnancy between 13 and 24 weeks of gestation

Author, year Country Design	GA	Intervention (I, *n*)/comparison (C, *n*)	Direct pain measure	Indirect pain measure	Limitations
Andersson,^19^ 2016 Sweden RCT	16	Bupivacaine PCB (I, 52)/saline PCB (C, 50)	*n* (%) with VAS > 7/10 ‐ I: 39 (75) ‐ C: 32 (65) ‐ RR: 1.1; 95% CI 0.9, 1.5	Morphine, median mg (IQR) I: 5 (1.3–10.5) C: 6 (1–10) RR: 0; 95% CI (−2, 2.5)	Did not meet projected sample size Groups differed at baseline
Winkler,^17^ 1997 Germany Comparative trial	21	Bupivacaine PCB (I, 10)/no PCB (C, 10)	Median (range) maximum VAS I: 8.5 (5–10) C: 7.0 (1–10) *P* = not significant	Meperidine, median mg (range) I: 100 (0–150) C: 50 (0–159) *P* = not significant	No randomisation, allocation concealment, blinding Underpowered
Tintara,^24^ 2018 Thailand RCT	18	Celecoxib (I, 28)/placebo (C, 28)	Mean (SD) VAS at TOP I: 4.6 (2.8) C: 7.3 (2.2) *P* = 0.01 Mean difference (SD) hourly VAS: −0.5 (0.15) *P* < 0.001	*n* (%) requiring morphine I: 12 (43) C: 12 (43) *P* = 1 Morphine, median mg (IQR) I: 3 (3–3.8) C: 7.5 (3–12) *P* = 0.08	Longer than recommended misoprostol dosing interval Blinding not described ‘VAS at TOP’ outcome is underpowered
Velipasaoglu,^20^ 2016 Turkey RCT	18	Diclofenac (I, 20)/acetaminophen (C1, 20)/hyoscine *N*‐butylbromide (C2, 20)	Mean (SD) VAS I: 4.0 (2.0) C1: 3.3 (1.3) C2: 3.2 (1.7) *P* = 0.35 Median (IQR) VAS at last miso I: 8 (4–9) C1: 7 (5–8.5) C2: 6 (4–9) *P* = 0.29	*n* (%) requiring meperidine I: 8 (40) C1: 5 (25) C2: 4 (20) *P* = 0.34	Blinding not described Assessment of outcome not clearly described Method to calculate single mean pain score not described Underpowered for three group analysis
Fiala,^21^ 2005 Sweden RCT	16	Diclofenac (I, 36)/paracetamol + codeine (C, 38)	Median (range) maximum VAS I: 7 (4–9) C: 7 (2–10) *P* = 0.7	*n* (%) requiring opioids I: 29 (81) C: 31 (82) *P* = 0.91 Opioids, median mg (IQR) I: 3.5 (0–25) C: 7 (0–53) *P* = 0.042	Inadequate blinding Six participants excluded post randomisation
Smith,^23^ 2016 Canada RCT	22	Epidural PCA (I, 17)/IV PCA (C, 20)	Mean (SD) maximum verbal pain score I: 4.2 (2.3) C: 5.9 (3.1 *P* = 0.07 Mean (SD) satisfaction score I: 8.4 (1.4) C: 7.8 (1.8) *P* = 0.31	*n* (%) requiring anxiolytics I: 0 (0) C: 3 (15) *P* = not significant	TOP regimen not described or consistent Blinding not possible Terminated early for low recruitment Three post‐allocation withdrawals
Maggiore,^22^ 2016 Italy RCT	19	Epidural–intermittent (I, 52)/epidural–continuous (C, 52)	All VAS comparisons: *P* > 0.05 Mean (SD) satisfaction VAS I: 8.4 (1.5) C: 7.3 (2.0) *P* = 0.005	Sufentanil, mean mcg (SD) I: 72.8 (29.2) C: 85.9 (34.1) *P* = 0.038	Lower limit of gestational age range not stated

Abbreviations: C, C1, C2, comparison, comparison 1, comparison 2; CI, confidence interval; GA, average gestational age in weeks; I, intervention; IQR, interquartile range; IV, intravenous; PCA, patient‐controlled analgesia PCB, paracervical block; RCT, randomised controlled trial; RR, risk ratio; SD, standard deviation; VAS, visual analogue score.

Four studies reported the results of surgical TOP (Figure 2; Tables 2 and S2), and included 349 participants from the USA and Pakistan.^13^, ^25^, ^26^, ^27^ Gestational ages were between 13 and 21 weeks; one study did not report an average gestational age.^27^ No studies reported indications for TOP. Three studies reported participants' pain using VAS,^13^, ^25^, ^26^ whereas one study asked participants to characterise pain verbally as none, mild, moderate or severe.^27^ One study reported participant satisfaction.^25^ In one study, women reported pain immediately post‐procedure;^26^ in all others, women reported pain upon awakening from general anaesthesia.^13^, ^25^, ^27^ Two studies reported the need for additional pain medication.^13^, ^26^

**Table 2:** Included studies of pain management for surgical termination of pregnancy between 13 and 24 weeks of gestation

Study, year Country Design	GA	Intervention (I, *n*)/Comparison (C, *n*)	Direct pain measure	Indirect pain measure	Limitations
Thaxton,^26^ 2018 USA Randomised non‐inferiority trial	13	Nitrous oxide (I, 19)/fentanyl + midazolam (C, 20)	Median (range) maximum VAS I: 6.6 (0.3–10) C: 1.7 (0–9.2) *P* = 0.001 Immediately postTOP I: 6.1 (1.2–10) C: 2.8 (0–9.4) *P* = 0.03	*n* (%) requiring fentanyl + midazolam ‐ I: 7 (37) ‐ C: 0 (0)	Maximum pain score assessed by recall at discharge Study stopped early based on predefined parameter Sample size not met
Micks,^25^ 2015 USA RCT	21	Sevoflurane (I, 80)/oxygen (C, 80)	Mean (SD) VAS at awakening I: 2.6 (2.3) C: 2.8 (2.2) *P* = 0.64 At discharge I: 2.2 (2.5) C: 2.0 (1.9) *P* = 0.77 Mean (SD) satisfaction VAS I: 9.4 (1.1) C: 9.3 (1.4) *P* = 0.5		
Lazenby,^13^ 2009 USA RCT	15	Bupivacaine PCB (I, 39)/no PCB (C, 33)	Mean (95% CI) VAS postprocedure I: 1.2 (0.5, 1.8) C: 1.6 (0.8, 2.4) *P* = 0.22 30 minutes post‐procedure I: 1.2 (0.5, 1.8) C: 2 (1.1, 2.8) *P* = 0.07 60 minutes post‐procedure I: 0.9 (0.2, 1.6) C: 0.8 (0.3, 1.3) *P* = 0.38	*n* (%) requiring pain medication ‐ I: 10 (27) ‐ C: 12 (34) ‐ *P* = 0.34	Only participants blinded
Siddiqui,^27^ 2007 Pakistan RCT	NR	Nalbuphine (I, 35)/tramadol (C, 35)	*n* (%) reporting no pain I: 28 (80) C: 18 (52) Mild pain I: 7 (20) C: 17 (48) Moderate or severe pain C: 0		Randomization, allocation, blinding not described Time of pain assessment not clear

Abbreviations: C, comparison; CI, confidence interval; GA, average gestational age in weeks; I, intervention; NR, not reported; PCB, Paracervical block; RCT, randomised controlled trial; SD, standard deviation; VAS, visual analogue score.

### Pain management during medical TOP

#### Local anesthesia

Two studies examined the effectiveness of paracervical block (PCB) for pain management.^17^, ^19^ One RCT compared PCB with 20 ml of 0.25% bupivacaine against saline placebo, administered 1 hour after the first misoprostol dose of a combined mifepristone and misoprostol medical TOP regimen.^19^ The proportion of women reporting severe pain (pain ≥ 7 on an 11‐point VAS: PCB = 75%, placebo = 65%, *P* = 0.292) and the use of additional pain medication (median morphine: PCB = 5 mg, placebo = 6 mg, *P* = 0.772) did not differ between groups. A second, nonrandomised trial compared bupivacaine PCB against no block in women undergoing medical TOP with gemeprost.^17^ This study found no difference in median VAS scores or in additional pain medication use (median meperidine: PCB = 100 mg, no PCB = 50 mg) between the two groups.

#### Nonsteroidal anti‐inflammatory drugs

Three RCTs compared nonsteroidal anti‐inflammatory drugs (NSAIDs) with other analgesics or with a placebo.^20^, ^21^, ^24^ In one trial,^21^ women receiving prophylactic diclofenac (100 mg) at misoprostol initiation as part of a mifepristone and misoprostol regimen reported the same maximum VAS pain scores as women receiving paracetamol plus codeine (1000/20 mg). In both groups, the same proportion of women required additional intravenous (IV) opioid pain medication (81 and 82%, respectively, *P* = 0.91),^21^ although the median dose of opioids used was lower in the NSAID group (3.5 and 7 mg respectively, *P* = 0.04). This finding was driven by the higher median opioid requirements for women with more advanced pregnancies (>15 weeks of gestation: NSAID = 5.5 mg, comparison = 10.5 mg, *P* = 0.02), but must be interpreted with caution as the gestational age subgroup analysis was inadequately powered. A second RCT compared the effectiveness of diclofenac (75 mg), paracetamol (500 mg) or hyoscine *N*‐butylbromide (HNBB, 10 mg), administered at the initiation of misoprostol in a misoprostol‐only regimen, and re‐administered throughout the TOP process.^20^ Investigators found no difference in the mean pain scores (NSAID = 4.0, acetaminophen = 3.3, HNBB = 3.2, *P* = 0.352), median pain score at last administration of misoprostol (NSAID = 8, acetaminophen = 7, HNBB = 6, *P* = 0.288) or proportion of women requiring additional analgesics (NSAID = 40%, acetaminophen = 25%, HNBB = 20%, *P* = 0.344). An additional RCT compared celecoxib (400 mg once) with placebo administered at the initiation of misoprostol in a misoprostol‐only regimen, finding that women reported lower pain scores during the TOP process (hourly mean difference = −0.5, *P* < 0.001) and at the time of expulsion (NSAID = 4.6, placebo = 7.3, *P* = 0.01).^24^ Pain at expulsion was reported only for women completing their TOP within 24 hours (NSAID = 57%, placebo = 68%, *P* = 0.64), rendering this outcome underpowered, however. In both groups, 43% of women required additional morphine, but the median dose was 3.0 mg in the NSAID group, compared with 7.5 mg in the placebo group (*P* = 0.08).

#### Regional anaesthesia

Two RCTs examined the use of patient‐controlled and/or regional analgesia.^22^, ^23^ The first compared epidural patient‐controlled analgesia (PCA) with bupivacaine plus fentanyl against IV PCA with fentanyl, finding no difference in the maximum mean pain scores (epidural PCA = 4.2, IV PCA = 5.9, *P* = 0.07), or satisfaction scores (epidural PCA = 8.4, IV PCA = 7.8, *P* = 0.31), between groups.^23^ The second study compared two strategies of epidural analgesia with levobupivacaine plus sufentanil in women undergoing medical TOP with gemeprost: programmed intermittent bolus or continuous infusion.^22^ The pain scores for both strategies were low and did not differ between groups (VAS < 2 at all assessments, *P* > 0.05). The intermittent bolus group reported higher mean satisfaction scores (intermittent bolus = 8.4, continuous infusion = 7.3, *P* = 0.005), whereas the continuous infusion group consumed more pain medication and reported more narcotic‐related side effects and nausea.

No differences were found between the intervention and the comparison groups in any of the studies reporting the following outcomes: the TOP success rate^20^, ^24^; the time interval between induction and TOP;^17^, ^19^, ^20^, ^21^, ^23^, ^24^ or the rate of adverse events.^19^, ^21^, ^22^, ^23^, ^24^

### Pain management during surgical TOP

Three of the four included studies examined the effect of adjuvant medications on post‐procedure pain in women receiving general anaesthesia or deep IV sedation.^13^, ^25^, ^27^ In a 2007 RCT,^27^ women who received IV nalbuphine at the time of induction of general anesthesia were more likely to have no post‐procedure pain than women who received tramadol (*P* = 0.022); no women in this study reported moderate or severe pain.^27^ A 2009 RCT found no differences in post‐procedure pain (PCB = 1.4, no PCB = 1.2, *P* = 0.3) or additional pain medication use (PCB = 10%, no PCB = 12%, *P* = 0.34) in women who received PCB with 10 ml of 0.5% bupivacaine in addition to general anaesthesia or deep IV sedation, compared with no PCB.^13^ A 2015 RCT assessed post‐procedure pain in women who received either inhaled sevoflurane or oxygen in addition to general anaesthesia.^25^ No differences in pain scores (sevoflurane = 2.6, oxygen = 2.8, *P* = 0.64) or satisfaction scores (sevoflurane = 9.4, oxygen = 9.3, *P* = 0.5) were found between the groups.

One additional RCT compared inhaled nitrous oxide (70% nitrous oxide/30% oxygen) against moderate sedation with IV fentanyl plus midazolam in women with gestations between 12 and 16 weeks.^26^ Pain scores were significantly higher in the nitrous oxide group immediately post‐TOP (nitrous oxide = 6.1, moderate sedation = 2.8, *P* = 0.03) and when participants were asked to recall their maximum pain (nitrous oxide = 6.6, moderate sedation = 1.7, *P* = 0.001). The study was stopped early when the proportion of women in the nitrous oxide group experiencing inadequate pain control requiring conversion to IV sedation exceeded a predefined maximum of 35%.

## Discussion

### Main findings

Although we found few comparable high‐quality studies assessing the effectiveness of pain management strategies for second‐trimester TOP, several findings are available to guide providers. For medical TOP between 13 and 24 weeks of gestation, studies indicate that: the prophylactic use of NSAIDs improve the experience of pain during the TOP process and decrease additional opioid requirements; regional analgesia appears to effectively manage pain; and PCB is ineffective. We found no studies assessing IM/IV opioids, anxiolytics or nonpharmacological treatment. For surgical TOP, general anaesthesia/deep IV sedation is effective and results in mild post‐procedure pain, with no additional benefit from adjuvant therapy, and nitrous oxide was ineffective. No studies assessed moderate IV sedation, IV/IM opioids, anxiolytics, PCB without sedation, NSAIDs or nonpharmacological treatment. Complications, adverse events and side effects were not affected by pain management strategies for medical or surgical TOP.

### Strengths and limitations

Pain management in TOP has previously been identified as a neglected issue,^28^ with limited evidence on which to base clinical practice, particularly in the second trimester;^10^, ^29^ however, in a marked improvement since our 2011 systematic review on this topic,^29^ we found multiple published RCTs assessing different treatments for women's TOP‐related pain. These studies used currently recommended TOP methods, validated tools to measure pain and robust statistical analysis. Three studies also included measures of a woman's satisfaction, perhaps a more relevant indicator of pain management given the complex physical, psychological and social factors that affect TOP‐related pain, and the challenges inherent in effectively treating and measuring pain.^22^, ^23^, ^25^, ^30^

This body of evidence suffers from several important limitations, however. For medical TOP, five of the seven included studies failed to calculate,^17^ incorrectly calculated,^20^ or did not meet the calculated sample size requirements,^19^, ^23^, ^24^ and only two described and implemented strategies for the blinding of participants and data collectors to treatment assignments.^19^, ^22^ For both medical and surgical TOP studies, the heterogeneity of pain management strategies and outcome reporting prevented a pooled analysis. Finally, the included studies typically examined resource‐intensive pain management strategies, which require specially trained staff, equipment and commodities, thereby limiting the applicability of the findings in low‐resource settings.

### Interpretation

The best available evidence suggests that women undergoing second‐trimester medical TOP should be offered either programmed intermittent bolus or continuous epidural analgesia, plus a prophylactic NSAID medication such as diclofenac, to minimise their pain. Where regional analgesia is unavailable or unacceptable, in addition to prophylactic NSAIDs we recommend regularly scheduled repeated doses of an appropriate parenteral opioid pain medication throughout the TOP process.^16^, ^31^ We found no studies examining different parenteral opioids or administration schedules upon which to base recommendations; however, a 2018 systematic review assessing parenteral opioids for pain management during term delivery, which in some ways approximates the labour‐like process of second‐trimester medical TOP, found that parenteral opioids provided some labour pain relief and moderate satisfaction.^32^ Although the authors concluded that there were insufficient data to recommend a best treatment, IV fentanyl, morphine and butorphanol performed better than meperidine, which performed better than tramadol or placebo.

General anaesthesia/deep IV sedation alleviated pain during surgical TOP, with no benefit of additional adjuvant therapy. Although deep IV sedation with propofol can safely be provided without intubation for women undergoing second‐trimester surgical TOP in the outpatient setting,^33^, ^34^, ^35^ both of these modalities require specially trained staff, specialised equipment and are resource intensive. An alternative is a multimodal approach to pain management during second‐trimester surgical TOP, including moderate IV sedation with opioids and anxiolytics, PCB and pre‐procedure NSAIDs.^16^ Given the lack of data specific to the second trimester these recommendations are based on studies of vacuum aspiration in early pregnancy, which demonstrate that: moderate IV sedation treats pain safely and effectively, and improves women's procedural satisfaction^30^, ^36^, ^37^; PCB decreases pain associated with cervical dilation and uterine aspiration^38^, ^39^, ^40^; and the pre‐procedure administration of oral or IM NSAIDs decreases pain, both during and after the procedure.^41^ Demonstrative data come from women in the comparison group of the Thaxton study, who received a combination of moderate IV sedation, PCB and pre‐procedure NSIADs, and who reported pain scores comparable with those of women who received general anaesthesia or deep sedation in other studies.^26^ Moderate IV sedation in combination with PCB is safe without pre‐procedure fasting or continuous IV access, and with appropriately trained staff and patient monitoring, up to 18 weeks of gestation.^42^, ^43^

## Conclusion

Without effective pain management, most women will experience intense pain during a second‐trimester TOP; indeed, many women in the included studies rated their pain as severe despite receiving an intervention intended to mitigate the pain. The management of a woman's pain during the TOP process continues to be an understudied area, despite the vital role that adequate pain management plays in providing high‐quality care.^9^, ^10^ Although the studies in this review explored a variety of different pain management strategies, we found limited evidence upon which to base recommendations for effective pain management during second‐trimester medical or surgical TOPs, particularly outside resource‐intensive settings. The examination of alternative pain management strategies using appropriate study designs, properly powered to show a difference between strategies, should be a priority. As pain intensifies with higher gestations, subgroup analyses by gestational age ranges should be planned. Studies should assess women's experience of pain using standardised measures to facilitate comparison and pooled analyses of results, as well as patient satisfaction with their pain management.^44^ The examination of the effectiveness of IV PCA, routine administration of IV or IM opioid medications during the medical TOP process, as well as the effectiveness of IM opioid medications for surgical TOP should be prioritised to meet the needs of low‐resource settings.

### Disclosure of interests

None declared. Completed disclosure of interests form available to view online as supporting information.

### Contribution to authorship

Both authors contributed equally to the design, planning, conduct and manuscript preparation for this article.

### Details of ethics approval

Not applicable.

### Funding

This work was paid for by Ipas.

## Supporting information


**Table S1**. Details of the included studies of pain management for medical termination of pregnancy between 13 and 24 weeks of gestation.

**Table S2**. Details of the included studies of pain management for surgical termination of pregnancy between 13 and 24 weeks of gestation.

**Appendix S1**. PubMed search strategy.

Supplementary Material

Supplementary Material
