# Supplementary material for: Pain management for medical and surgical termination of pregnancy between 13 and 24 weeks of gestation: a systematic review
Source: BJOG. 2020 Apr 3;127(11):1348–57. doi: 10.1111/1471-0528.16212 (PMC7539983; doi:10.1111/1471-0528.16212)
Supplement: Supplementary file 1 — Table S1. Details of the included studies of pain management for medical termination of pregnancy between 13 and 24 weeks of gestation. [file BJO-127-1348-s001.pdf]

Table S1. Details of included studies of pain management for medical abortion between 13 and 24 weeks gestation

Study<br>Date<br>Country<br>Design	Inclusion criteria	Abortion regimen	Intervention/ Comparison	Results
Andersson, 2016<br>Sweden<br>RCT	N=102 women ≥ 18 years, with gestations between 13-22 weeks	Day 0: Mifepristone (dose not specified)<br><br>Day 2: Repeat doses of misoprostol (dose and interval not specified)	Intervention (n=52): paracervical block with 20mL 0.25% bupivacaine (2 injection sites, deep injection) administered 1 h after first misoprostol dose<br><br>Comparison (n=50): paracervical block with 20mL normal saline (2 injection sites, deep injection) administered 1 h after first misoprostol dose<br><br>All: NSAID (not specified) and paracetamol (dose not specified). Morphine IV as needed.	**Direct measurement of pain**<br>N (%) with pain score > 7 on 10cm VAS<br>Intervention: 39 (75)<br>Comparison: 32 (65)<br>Risk ratio: 1.1 (95% CI 0.9, 1.5)<br>P=0.292<br><br>**Indirect measurement of pain**<br>Median mg (IQ range) of additional morphine<br>Intervention: 5 (1.3-10.5)<br>Comparison: 6 (1-10)<br>Risk ratio: 0 (95% CI -2, 2.5)<br>P=0.772<br><br>**Safety and Side Effects**<br>No differences in side effects between two groups. One adverse event (sensory loss and weakness 30 mins after bupivacaine PCB, resolved)<br><br>**Induction to abortion interval**<br>Median minutes (IQ range)<br>Intervention: 435 (320-748)<br>Comparison: 398 (260-540)<br>Risk ratio: 80 (95% CI -5, 180)<br>P=0.075
Winkler, 1997<br>Germany<br>Comparative Trial	N=20 women between 16-42 year of age with pregnancies between 16-23 gestational weeks seeking abortion for fetal indications	Gemeprost 1mg PV q6hr with IV oxytocin augmentation as needed	Intervention (n=10): Paracervical block with 10mL of 0.5% bupivacaine (2 injection points)<br><br>Comparison (n=10): no paracervical block<br><br>All: Meperidine 50mg IV and butylscopalamine 10mg PR with additional doses as needed	**Direct measurement of pain**<br>Maximum pain score on 11-point VAS, median (range)<br>Intervention: 8.5 (5-10)<br>Comparison: 7.0 (1-10)<br>P=not significant<br><br>**Indirect measurement of pain**<br>Median mg (range) of additional meperidine<br>Intervention: 100 (0-150)<br>Comparison: 50 (0-159)<br>P=not significant<br><br>**Induction to abortion interval**<br>Median hours (range)<br>Intervention: 13 (8-36)<br>Comparison: 20 (8-44)<br>P=not significant

Tintara, 2018<br>Thailand<br>RCT	N=56 women with gestations between 14-24 weeks	Misoprostol SL 400mcg every 6 hours	Intervention (n=28): celecoxib 400mg po x 1<br><br>Comparison (n=28): placebo<br><br>All: morphine 3mg IV every 3 hours if pain score ≥ 7	**Direct measurement of pain**<br>Mean (SD) pain score on 10cm VAS at time of complete abortion<br>Intervention: 4.6 (2.8)<br>Comparison: 7.3 (2.2)<br>P=0.01<br><br>Mean difference (SD) in hourly pain score: -0.5 (0.15), p<0.001<br><br>**Indirect measurement of pain**<br>N (%) requiring additional IV morphine<br>Intervention: 12 (43)<br>Comparison: 12 (43)<br>P=1<br><br>Median mg (IQ range) of additional IV morphine<br>Intervention: 3 (3-3.8)<br>Comparison: 7.5 (3-12)<br>P=0.08<br><br>**Safety and Side Effects**<br>No differences in rates of fever, chills, nausea/vomiting,itching, or diarrhea between groups. No adverse events reported.<br><br>**Induction to abortion interval**<br>Median hours (IQ range)<br>Intervention: 19.4 (10.8-40.4)<br>Comparison: 15.2 (10-31.1)<br>P=0.43<br><br>**Abortion success**<br>N (%) achieving abortion within 24 hours<br>Intervention: 16 (57)<br>Comparison: 19 (68)<br>P=0.64
Velipasaoglu, 2016<br>Turkey<br>RCT	N=60 women with gestations between 13-22 weeks with an indication for abortion	Misoprostol 400mcg PV every 4hr for 48 hours	Acetaminophen (n=20): 500mg PO at first miso, then every 6hr x 2, then every 12hr<br><br>Diclofenac (n=20): 75mg PO at first miso, then every 6hr x 2, then every 12hr	**Direct measurement of pain**<br>Mean (SD) pain scores on 10-cm VAS<br>Acetaminophen: 3.31 (1.34)<br>Diclofenac: 4.02 (2.02)<br>HnBB: 3.17 (1.65)<br>P=0.352<br><br>Median (IQ range) pain score at final misoprostol administration<br>Acetaminophen: 7 (5-8.5)

			Hyoscine N-butylbromide (HnBB, n=20): 10mg po at first miso, then q6hr x 2, then q12hr<br><br>All: Meperidine IV as needed	Diclofenac: 8 (4-9)<br>HnBB: 6 (4-9)<br>P=0.288<br><br>**Indirect measurement of pain**<br>N (%) requiring additional IV meperidine<br>Acetaminophen: 5 (25)<br>Diclofenac: 8 (40)<br>HnBB: 4 (20)<br>P=0.344<br><br>**Induction to abortion interval**<br>Median (IQ range) hours<br>Acetaminophen: 21.5 (11.25-32)<br>Diclofenac: 28 (20.25-34.5)<br>HnBB: 16 (12.5-27.5)<br>P=0.611<br><br>**Abortion success**<br>N (%) achieving abortion within 24 hours<br>Acetaminophen: 11 (55)<br>Diclofenac: 11 (55)<br>HnBB: 14 (70)<br>p=0.535
Fiala, 2005<br>Sweden<br>RCT	N=74 women with singleton pregnancies between 13-22 weeks	Day 0: Mifepristone 600mg PO x 1<br><br>Day 2-3: Misoprostol 800mcg PV x1, then 400mcg PO every 3 hours	Intervention (n=36): diclofenac 100mg PO x 1<br><br>Comparison (n=38): paracetamol 1000mg +codeine 20mg PO x 1<br><br>All: paracetamol, codeine, IV opiates or paracervical block as needed	**Direct measurement of pain**<br>Median (range) maximum pain score on 10cm VAS<br>Intervention: 7 (4-9)<br>Comparison: 7 (2-10)<br>P=0.7<br><br>**Indirect measurement of pain**<br>N (%) requiring additional IV opiates<br>Intervention: 29 (81)<br>Comparison: 31 (82)<br>P=0.91<br><br>Median mg (IQ range) of additional IV opiates<br>Intervention: 3.5 (0-25)<br>Comparison: 7 (0-53)<br>P=0.042<br><br>N (%) requiring additional oral pain medications<br>Intervention: 9 (25)<br>Comparison: 16 (42)<br>P=0.12<br><br>N (%) requiring additional paracervical block<br>Intervention: 4 (11)<br>Comparison: 2 (5)<br>P=0.42

				**Safety and Side Effects**<br>No statistically significant differences in fever, nausea, vomiting and diarrhea. No differences in blood loss. No serious complications reported.<br><br>**Induction to abortion interval** (for those with abortion interval <24 h)<br>Median hours (range)<br>Intervention: 5.4 (2.1-23.2)<br>Comparison: 6.5 (2.8-22.0)<br>P=0.13
Smith, 2016<br>Canada<br>RCT	N=37 women > 18 years with gestations between 12-23+6 weeks	"Misoprostol per physician preference"	Intervention (n=17): Epidural patient-controlled analgesia with bupivacaine and fentanyl (5mL bolus of 0.0625% bupivacaine + fentanyl 2mg/mL with 10 minute lockout, then infusion of 10mL/hr)<br><br>Comparison (n=20): IV patient-controlled analgesia with fentanyl (25-50mcg bolus with 3-6 minute lockout)	**Direct measurement of pain**<br>Mean (SD) maximum pain score on 11-point verbal numeric pain score during first 24 hours<br>Intervention: 4.2 (2.3)<br>Comparison: 5.9 (3.1)<br>P=0.07<br><br>Mean (SD) satisfaction score on 11-point verbal numeric scale<br>Intervention: 8.4 (1.4)<br>Comparison: 7.8 (1.8)<br>P=0.31<br><br>**Indirect measurement of pain**<br>N (%) requiring additional anxiolytic medication<br>Intervention: 0 (0)<br>Comparison: 3 (15)<br>P=not significant<br><br>**Safety and Side Effects**<br>No statistically significant differences in rates of retained products of conception or surgical evacuation of the uterus. No statistically significant differences in side effects (nausea, vomiting, pruritis, sedation, hypotension or need for bladder catheterization). One participant in the intervention group received a transfusion.<br><br>**Induction to abortion interval**<br>No statistically significant differences in induction to abortion interval (data not shown)

Maggiore, 2016<br>Italy<br>RCT	N=104 women ≥ 18 years with "second trimester" pregnancies up to 24 weeks gestation (mean 19 weeks), request for analgesia with a baseline pain score ≥ 30mm	Gemeprost 1mg PV q3hr up to 5 doses, repeated for a second cycle if needed<br><br>In women with uterine scar, gemeprost 1mg PV q6hr up to 5 doses for one cycle only	Intervention (n=52): Programmed intermittent epidural bolus technique: 10mL of 0.0625% levobupivacaine + sufentanil 0.5mcg/mL every 1 hour<br><br>Comparison (n=52): Continuous epidural infusion technique with 0.0625% levobupivacaine and sufentanil 0.5mcg/mL at a rate of 10mL/hr	**Direct measurement of pain**<br>"Visual analogue pain scores were similar between the study groups and they were at all times <20mm at the hourly assessments (p>0.05 for all comparisons)."<br><br>Mean (SD) satisfaction score on 10cm VAS<br>Intervention: 8.4 (1.5)<br>Comparison: 7.3 (2.0)<br>P=0.005<br><br>**Indirect measurement of pain**<br>Total consumption of levobupivacaine (mg), mean (SD)<br>Intervention: 91 (36.5)<br>Comparison: 107.3 (42.6)<br>P=0.038<br><br>Total consumption of sufentanil (mcg), mean (SD)<br>Intervention: 72.8 (29.2)<br>Comparison: 85.9 (34.1)<br>P=0.038<br><br>**Safety and Side Effects**<br>N (%) with at least one narcotic-related side effect<br>Intervention: 21 (40.4)<br>Comparison: 33 (63.5)<br>P=0.031<br><br>N (%) with nausea<br>Intervention: 7 (13.5)<br>Comparison: 18 (34.6)<br>P=0.022<br><br>No statistically significant differences in vomiting, pruritis, sedation or respiratory depression. No differences in epidural related adverse events (hypotension, dural puncture, neurologic complications, shivering) or abortion related adverse events (hemorrhage, transfusion, perforation).

V=intravenous; IQ=interquartile; Mg=milligram; Mcg=microgram; PO=per os; PV=per vaginum; PR=per rectum; VAS=visual analogue scale
