# Supplementary material for: Pain management for medical and surgical termination of pregnancy between 13 and 24 weeks of gestation: a systematic review
Source: BJOG. 2020 Apr 3;127(11):1348–57. doi: 10.1111/1471-0528.16212 (PMC7539983; doi:10.1111/1471-0528.16212)
Supplement: Supplementary file 2 — Table S2. Details of the included studies of pain management for surgical termination of pregnancy between 13 and 24 weeks of gestation. [file BJO-127-1348-s002.pdf]

Table S2. Details of included studies of pain management for surgical abortion between 13 and 24 weeks gestation

Study, year<br>Country<br>Design	Inclusion criteria	Intervention/ Comparison	Results
Thaxton, 2018<br>USA<br>Randomized noninferiority trial	N=39 women with gestations between 12-16 weeks	Intervention (n=19): inhaled nitrous oxide (70% nitrous/30% oxygen)<br><br>Comparison (n=20): IV fentanyl (100mcg) + IV midazolam (2mg)<br><br>All: paracervical block with 20mL buffered lidocaine, ibuprofen 600mg po x 1, and IV fentanyl and/or IV midazolam as needed	**Direct measurement of pain**<br>Median (range) pain score on 10cm VAS<br>*Anticipated pain*<br>Intervention: 4.9 (1.0-9.9)<br>Comparison: 5.4 (1.0-9.9)<br>P=0.63<br><br>*Baseline*<br>Intervention: 0.3 (0-5.0)<br>Comparison: 0.2 (0-7.5)<br>P=0.77<br><br>*Immediately postabortion*<br>Intervention: 6.1 (1.2-10)<br>Comparison: 2.8 (0-9.4)<br>P=0.03<br><br>*Maximum pain (by recall)*<br>Intervention: 6.6 (0.3-10)<br>Comparison: 1.7 (0-9.2)<br>P=0.001<br><br>**Indirect measurement of pain**<br>N (%) requiring additional IV fentanyl and midazolam<br>Intervention: 7/19 (37)<br>Comparison: 0/20 (0)<br><br>**Safety and Side Effects**<br>No adverse events reported.
Micks, 2015<br>USA<br>RCT	N=160 women with gestations between 18-24 weeks	Intervention (n=80): Inhaled sevoflurane plus standard general anesthesia protocol (IV propofol, IV midazolam, IV fentanyl and inhaled nitrous oxide)<br><br>Comparison (n=80): Inhaled oxygen plus standard general anesthesia protocol	**Direct measurement of pain**<br>Mean (SD) pain score on 10cm VAS<br>*At awakening*<br>Intervention: 2.6 (2.3)<br>Comparison: 2.8 (2.2)<br>P=0.64<br><br>*At discharge*<br>Intervention: 2.2 (2.5)<br>Comparison: 2.0 (1.9)<br>P=0.77<br><br>Mean (SD) satisfaction on 10cm VAS<br>Intervention: 9.4 (1.1)<br>Comparison: 9.3 (1.4)<br>P=0.51<br><br>**Safety and Side Effects**<br>No statistically significant differences in side effects or complications, including blood loss.

Lazenby, 2009<br>USA<br>RCT	N=72 women with gestations between 5-20 weeks (mean 15 weeks)	Intervention (n=39): paracervical block with 10mL 0.5% bupivacaine<br><br>Comparison (n=33): no paracervical block<br><br>All: General anesthesia (multiple modalities) or IV deep sedation with propofol + midazolam + fentanyl; additional ketorolac, meperidine or morphine if needed	**Direct measurement of pain**<br>Mean (95% CI) pain score measured on 10cm VAS<br>*Preprocedure*<br>Intervention: 1.4 (0.8, 2.1)<br>Comparison: 1.2 (0.6, 1.9)<br>P=0.3<br><br>*Immediately postprocedure*<br>Intervention: 1.2 (0.5, 1.9)<br>Comparison: 1.6 (0.8, 2.4)<br>P=0.22<br><br>*30 minutes postprocedure*<br>Intervention: 1.2 (0.5, 1.8)<br>Comparison: 2.0 (1.1, 2.8)<br>P=0.07<br><br>*60 minutes postprocedure*<br>Intervention: 0.9 (0.2, 1.6)<br>Comparison: 0.8 (0.3, 1.3)<br>P=0.38<br><br>*Before discharge*<br>Intervention: 0.5 (0.2, 0.9)<br>Comparison: 0.3 (0.1, 0.6)<br>P=0.2<br><br>**Indirect measurement of pain**<br>N (%, 95% CI) requiring additional pain medication<br>Intervention: 10 (27, 13.9, 40)<br>Comparison: 12 (34, 16.7, 54.5)<br>Relative Risk: 1.26 (0.62, 2.55, p=0.34)<br><br>**Safety and Side Effects**<br>No adverse events reported.
Siddiqui, 2007<br>Pakistan<br>RCT	N=70 women with gestations between 12-14 weeks	Intervention (n=35): IV Nalbuphine 0.1mg/kg<br><br>Comparison (n=35): IV Tramadol 1.5mg/kg<br><br>All: General anesthesia (propofol + succinylcholine)	**Direct measurement of pain**<br>N (%) of women reporting no, mild, moderate or severe pain<br>*No pain*<br>Intervention: 28 (80)<br>Comparison: 18 (52)<br><br>*Mild*<br>Intervention: 7 (20)<br>Comparison: 17 (48)<br><br>*Moderate*<br>Intervention: 0<br>Comparison: 0<br><br>*Severe*<br>Intervention: 0<br>Comparison severe: 0

IV=intravenous; CI=confidence interval; Kg=kilogram; Mg=milligram; Ml=milliliter; PO=per os; VAS=visual analogue scale
