# Supplementary material for: Pain management for medical and surgical termination of pregnancy between 13 and 24 weeks of gestation: a systematic review
Source: BJOG. 2020 Apr 3;127(11):1348–57. doi: 10.1111/1471-0528.16212 (PMC7539983; doi:10.1111/1471-0528.16212)
Supplement: Supplementary file 3 — Appendix S1. PubMed search strategy. [file BJO-127-1348-s003.pdf]

Appendix S1. PubMed search strategy

(("Abortion, Induced"[Mesh] OR ("abortion"[All Fields] AND "induced"[All Fields]) OR "induced abortion"[All Fields] OR "abortion"[All Fields] OR "termination of pregnancy"[All Fields] OR "pregnancy termination"[All Fields]) AND ("Pregnancy Trimester, Second"[MeSH Terms] OR second trimester OR second-trimester OR midtrimester OR mid-trimester OR "dilatation and evacuation"[All Fields] OR "dilation and evacuation"[All Fields])) AND ("Pain Management"[Mesh] OR "Anesthesia and Analgesia"[Mesh] OR "Complementary Therapies"[Mesh] OR "pain management"[All Fields] OR "pain control"[All Fields] OR "pain med*"[All Fields] OR "analgesia"[All Fields] OR "anesthesia"[All Fields] OR "sedation"[All Fields] OR "pain"[All Fields])
